# A rapid and accurate method for estimating the erythrocyte sedimentation rate using a hematocrit-corrected optical aggregation index

**DOI:** 10.1371/journal.pone.0270977

**Published:** 2022-07-12

**Authors:** Makoto Higuchi, Nobuo Watanabe

**Affiliations:** 1 Functional Control Systems Course, Graduate School of Engineering and Science, Shibaura Institute of Technology, Saitama, Japan; 2 Ogino Memorial Laboratory, Nihon Kohden Corporation, Tokyo, Japan; University of Glasgow, UNITED KINGDOM

## Abstract

Although both the erythrocyte sedimentation rate (ESR) and optically measured erythrocyte aggregation parameters are affected by the hematocrit, this interaction is not considered by the method used to estimate ESR that considers aggregation parameters. In this study, we investigated the relationship between the ESR obtained by the Westergren method and that obtained with an aggregation parameter, namely, the aggregation index (AI) of multiple hematocrit values and fibrinogen-spiked samples with an analysis time of 5–60 s, and attempted to develop a rapid and accurate ESR estimation method. The AIs obtained from 5- and 10-s optical measurements with a fixed hematocrit were highly correlated with the erythrocyte sedimentation velocity. Furthermore, the rate of the AI increase with an increasing hematocrit was not significantly affected by the fibrinogen concentration at these measurement times. On the basis of these results, we defined the hematocrit-corrected aggregation index (HAI). The exponential function of the HAI obtained from the 5-s measurement agreed well with the sedimentation velocity calculated to eliminate the effect of hindered settling, and the HAI and hematocrit could be used to calculate the time constant of the sedimentation curve with a linear regression equation. The ESR value at 1 h was calculated based on the modified Stokes’ law and the HAI obtained from the 5-s measurement and showed an excellent correlation (*R* = 0.966) with the ESR value obtained by the Westergren method over a wide range of hematocrit and fibrinogen concentrations.

## Introduction

The erythrocyte sedimentation rate (ESR) is elevated in various diseases, including infections, pneumonia, vasculitis, and rheumatoid arthritis [1–3]. Because it can be measured easily and inexpensively, the ESR has long been used in clinical settings worldwide as a useful marker of inflammation. The classic and current international reference method for determining the ESR is the Westergren method [4, 5].

The erythrocyte sedimentation curve comprises three phases for each measurement time [6]. In the first phase (lag phase), erythrocytes dispersed in plasma form one-dimensional coin stacks (*rouleaux*). *Rouleaux* form aggregates by gathering in two- to three-dimensions over time, and sedimentation of the erythrocyte–plasma interface occurs after a certain delay. At this time, the aggregate size increases according to the plasma concentration of fibrinogen or globulin [1, 7] and decreases as the hematocrit (Ht) increases [6, 8–10]. The main phase of the ESR is the second (sedimentation) phase, in which the sedimentation rate becomes maximum and almost constant. In this phase, the ESR can be described through application or modification of Stokes’ law [11], which is an equation for calculating the settling speed of a single particle [6, 12, 13]. According to Stokes’ law, the settling speed of a particle is proportional to the square of its radius and the difference in the density between the particle and the solution and is the inverse of the viscosity of the solution [11]. Stokes’ law was modified to include the hindered settling effect, which is the interference of upward flow on the sedimentation velocity determined by the Ht [12–14]. In the third (packing) phase, the sedimentation rate is reduced by the deposition of erythrocytes at the bottom of the tube. Finally, the sedimentation distance converges on the value corresponding to the volume ratio of blood cells and plasma over time [6]. Thus, erythrocyte sedimentation is a complex phenomenon that is greatly influenced by the concentration of plasma proteins and Ht.

One disadvantage facing the clinical use of the Westergren method is its long test duration of 1 h. To shorten the test duration by up to 50%, the acceleration of sedimentation velocity has been studied by using an inclined tube (Boycott effect) [15, 16]. Moreover, much more rapid ESR measurement methods have been reported [17, 18] using aggregation parameters calculated from a syllectogram, which is the transmitted or reflected light intensity waveform caused by the formation of erythrocyte aggregates [19]. In a syllectogram, a rapid increase in light transmittance corresponds to two-dimensional *rouleaux* formation, which occurs within 1–5 s [20, 21]. In contrast, the slow increase in light transmittance from 10 to 60 s is related to the growth of three-dimensional erythrocyte aggregates. Multiple aggregation parameters can be calculated from a syllectogram to easily analyze erythrocyte aggregation, and the relationships between the aggregation parameters and various biomarkers and disease have been studied [22–24].

However, the ESR value obtained using aggregation parameters does not agree with the ESR value generated using the Westergren method (WG ESR) [25, 26], and the International Council for Standardization in Haematology recommends that the differences in the methods be considered [5]. There are two reasons for this discrepancy. The first reason is that the Ht affects all three sedimentation phases [6, 10] and the syllectogram [20, 27, 28] through different mechanisms and at different magnitudes. In other words, the syllectogram alone cannot distinguish the effects of erythrocyte aggregation, hindered settling, and packing. Therefore, to estimate the ESR using aggregation parameters, the method needs to consider the effects of the Ht on the syllectogram, hindered settling, and maximum settling distance. The second reason is that the relationship between the aggregation parameters and ESR depends greatly on the syllectogram measurement time [18, 28]. Hence, the effect of the Ht and the syllectogram measurement time should both be considered. Indeed, several high-speed ESR assays have been proposed, but their practicality has not been fully demonstrated because the measurement time needs to be even shorter for clinical use and they have not been sufficiently validated for widely varying concentrations of the Ht and plasma proteins [18, 29].

In this study, we investigated the relationship between the ESR obtained by the Westergren method and an aggregation parameter, namely, the aggregation index (AI) at multiple Ht values and in fibrinogen-spiked samples with a measurement time of 5–60 s. Based on the result, we attempted to estimate the WG ESR at 1 h (WG ESR_1h_) using the modified Stokes’ law and the Ht-corrected AI.

## Materials and methods

### Sedimentation theory and estimation of the sedimentation curve

Puccini *et al*. used Eq (1) to express the settling distance *h*_*p*_ (*t*) at time *t* as an equation describing the sigmoidal sedimentation curve [30].


$$h_{p}(t)=h_{\infty }[1-\frac{1}{{\left(t/t_{50}\right)}^{\beta }+1}]$$ (1)

Here, *h*_*∞*_ is the maximum settling distance after a sufficiently long time, *t*_*50*_ is the time when the settling distance is half of *h*_*∞*_, and *β* is a coefficient. This equation has a good fit but is not suitable for ESR estimation over short time frames because each coefficient has no physical meaning and no generality and because the sedimentation curve needs to be observed for a long time to obtain *h*_*∞*_. Stokes’ law [11], shown as Eq (2), has been commonly used to explain the ESR.


$$V_{e}=\frac{2(\rho_{e}-\rho_{p})g}{9\mu_{p}}R_{ef}^{2}$$ (2)

Here, *ρ*_*e*_ is the erythrocyte density [kg/m^3^], *ρ*_*p*_ is the plasma density [kg/m^3^], *g* is the gravity acceleration [m/s^2^], *R*_*ef*_ is the effective erythrocyte radius [m], and *μ*_*p*_ is the plasma viscosity [Pa·s]. However, the sedimentation velocity *V*_*e*_ is slower in a dense particle population due to the hindered settling effect caused by the interaction among the particles. The hindered settling effect is defined as Eq (3), and the modified Stokes’ law is described by Eq (4), but these equations do not take into account erythrocyte aggregation.


$$\varphi (Ht)=\frac{V_{e}}{V_{s}}$$ (3)

$$V_{e}=\frac{2(\rho_{e}-\rho_{p})g}{9\mu_{p}}{\varphi (Ht)R}_{ef}^{2}$$ (4)

Oka described *V*_*e*_ in Eq (5) using the effective radius *R*_*agg*_ [m] of particles in the presence of erythrocyte aggregation, shown in Eq (6) [12].


$$V_{e}=\frac{2(\rho_{e}-\rho_{p})g}{9\mu_{p}}\varphi (Ht)R_{agg}^{2}$$ (5)

$$R_{agg}=R_{ef}\{1+\alpha (1-e^{-\frac{t}{\lambda }})\}$$ (6)

Here, *α* is the dimensionless size parameter of erythrocyte aggregation and *λ* is the time constant [s]. Therefore, in the initial phase of sedimentation, the sedimentation velocity at time *t* is expressed as Eq (7):

$$V_{e}=\frac{2(\rho_{e}-\rho_{p})g}{9\mu_{p}}\varphi (Ht){\left[R_{ef}\{1+\alpha (1-e^{-\frac{t}{\lambda }})\}\right]}^{2}$$ (7)

The increase in the erythrocyte aggregation size converges with time and, after a certain amount of time, the erythrocytes reach constant sedimentation velocity, as described by Eq (8).


$$V_{e}=\frac{2(\rho_{e}-\rho_{p})g}{9\mu_{p}}\varphi (Ht){\left[R_{ef}(1+\alpha )\right]}^{2}$$ (8)

Here, as discussed by Mayer, Eq (8) can be integrated to obtain the sedimentation distance *h*_*1*, *2*_ [m] from the lag phase to the constant sedimentation velocity phase, giving Eq (9) [13]:

$$h_{1,2}=\int_{0}^{t}V_{e}dt=\frac{2(\rho_{e}-\rho_{p})g}{9\mu_{p}}\varphi (Ht)R_{agg}^{2}{\left[{\left(1+\alpha \right)}^{2}t+\alpha \lambda \{(2\alpha +2)e^{-\frac{t}{\lambda }}-\frac{\alpha }{2}e^{-\frac{2t}{\lambda }}-\frac{3}{2}\alpha -2\}\right]}^{2}$$ (9)

Various functions have been reported for *φ* from experimental data [31, 32]. Here, we will use the equation reported by Richardson and Zaki, shown as Eq (10) [32].


$$\varphi (Ht)={\left(1-Ht\right)}^{n}$$ (10)

For low Reynolds number conditions, such as the ESR, *n* = 4.65. Here, the upper limit of *t* in the above equation is defined as the transition time τ from the constant sedimentation velocity phase to the packing phase. This transition time τ can be calculated by Eq (11), reported by Mayer [13].


$$\tau =10.317V_{e}^{-0.57}$$ (11)

The settling distance *h*_*3*_ [m] after the transition time τ can additionally be described by Eq (12), also reported by Mayer. Here, *h*_*∞*_ is reported to be related to the Ht and *α*, as shown in Eq (13) [13].


$$h_{3}(t)=h_{\infty }-[h_{\infty }-h(\tau )]exp[\frac{V_{e}(\tau )}{h_{\infty }-h(\tau )}(\tau -t)]$$ (12)

$$h_{\infty }=102(1-0.8Ht)\alpha^{0.2}$$ (13)

From the above, a sigmoidal erythrocyte sedimentation curve can be calculated from *Ht*, *V*_*e*_, *α*, and *λ*. For the calculation, we used the following literature values: *ρ*_*e*_ = 1100 kg/m^3^ [33], *ρ*_*p*_ = 1025 kg/m^3^ [34], *R*_*ef*_ = 3.084×10^−6^ m [13], and *μ*_*p*_ = 1.64 × 10^−3^ Pa·s [35].

### Preparation of fibrinogen-spiked blood

Venous blood from two healthy volunteers was collected in K2-EDTA tubes (Sekisui Medical Corporation, Tokyo, Japan), and plasma was separated by centrifugation at 1,697 × *g* for 10 min. Three levels of spiked samples (Fib+, Fib++, and Fib+++) were prepared by adding 0.4, 0.8, and 1.2 g/dL bovine fibrinogen (Sigma-Aldrich, St. Louis, MO), respectively, to the plasma. Normal or spiked plasma samples and the separated blood cells were mixed, and four different Ht (0.25, 0.30, 0.35, and 0.40) blood samples were prepared for each fibrinogen level. The experiments were conducted in accordance with the Declaration of Helsinki with the approval of the ethics committee of Nihon Kohden Corporation (approval no. ER67-09). Written informed consent was obtained from the volunteers.

### Determination of the sedimentation parameters for the Westergren method

ESR was measured by the Westergren method in accordance with Clinical and Laboratory Standards Institute guideline H02-A5 [36]. The prepared blood sample and a 3.2% sodium citrate solution were mixed in a 4:1 ratio. The mixed sample was aspirated into a vertically installed plastic Westergren tube (full-scale length, 200 mm; inner diameter, 2.55 mm; Terumo Corporation, Tokyo, Japan). The sedimentation distance of the blood cells was read visually in 0.5 mm at 1-min intervals until apparent sedimentation was observed and every 1 or 10 min thereafter for up to 60 min. The ambient measurement temperature was 21 ± 1°C. The sedimentation value at 1 h was adopted as the WG ESR_1h_. The sedimentation velocity *V*_*e*_ was determined as the slope of the linear regression line by the least-squares method in the range of sedimentation times where the settling velocity was constant. The analysis time was between 15 and 60 min for most samples but was adjusted as appropriate to extract the range of constant velocity. The size parameter *α* of erythrocyte aggregation can be calculated from *V*_*e*_ and Eq (8). The time constant *λ* was determined by curve fitting with the sedimentation curve described in the above section. *λ* values with a sedimentation velocity <0.1 mm/min were excluded from the analysis because the effect of the resolution error of the scale is relatively high. Based on Eqs (3) and (8), *V*_*s*_, which is the sedimentation velocity of an erythrocyte aggregate divided by the hindered settling effect, was calculated by Eq (14):

$$V_{s}=\frac{V_{e}}{{\left(1-Ht\right)}^{n}}$$ (14)

### Erythrocyte aggregation and complete blood count measurements

**Fig 1:**
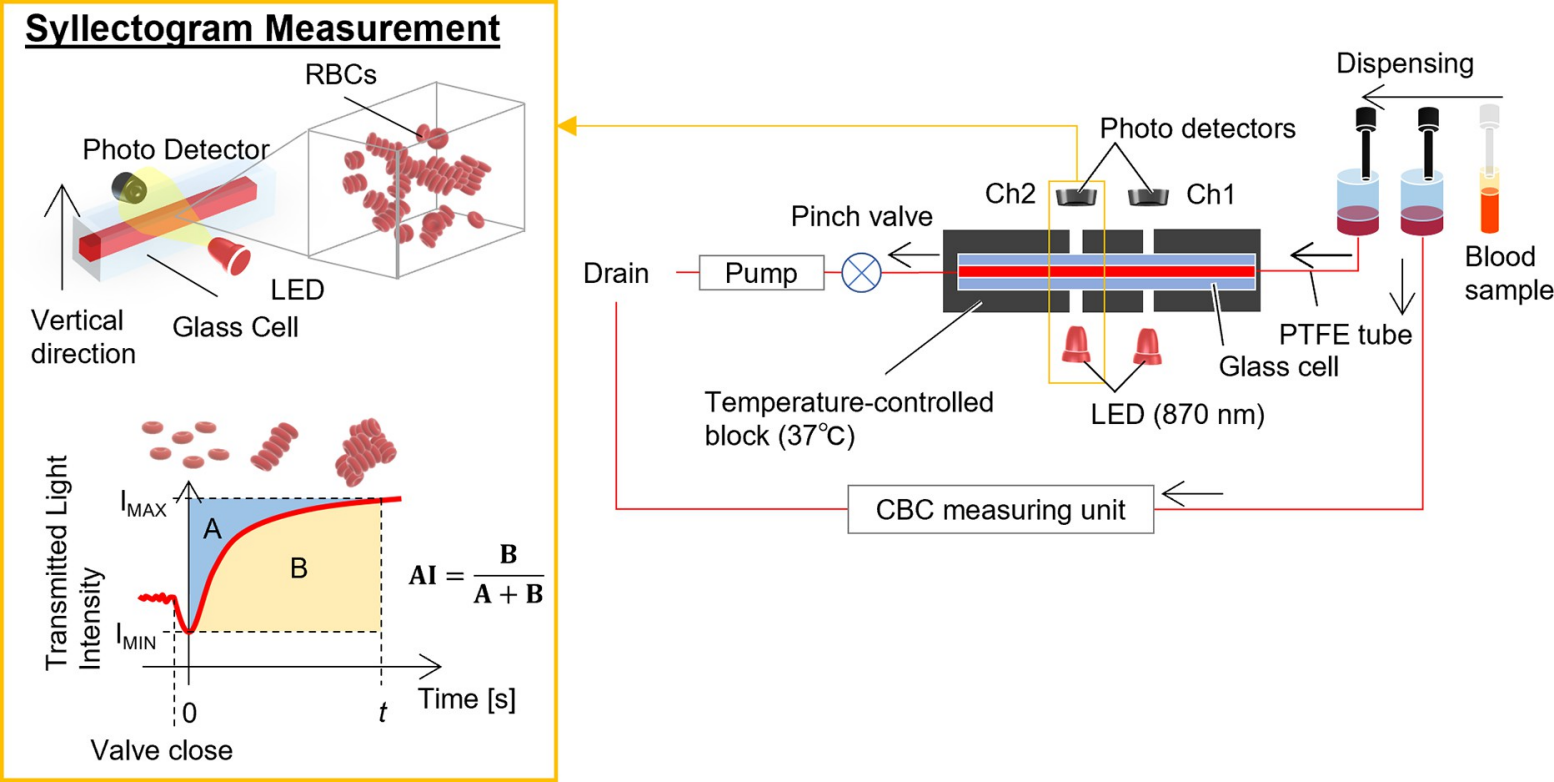
Experimental apparatus and aggregation parameters. The blood sample is aspirated by the nozzle, dispensed into a reservoir, and then moved by a pump to the erythrocyte aggregation measurement section, which is maintained at 37°C. The flow is stopped abruptly by the valve, and transmitted light intensity is measured. The transmitted light is measured by LEDs and photodiodes placed horizontally against the blood in the measuring cell. The time at which the transmitted light intensity becomes the minimum value (I_MIN_) after sudden flow cessation was defined as the starting time (t = 0). Aggregation index (AI): ratio of area A to the total of areas A and B. Area A is defined as the enclosed area between the syllectogram and I_MIN_. Area B is defined as the area between the syllectogram and I_MAX_. At the same time, blood dispensed into the reservoir for the CBC is measured by the CBC measurement section.

A schematic diagram of the analyzer (MEK-1305; Nihon Kohden Corporation, Tokyo, Japan) is shown in Fig 1. The analyzer aspirated 80 μL of blood and dispensed 60 μL of the sample into the reservoir connected to the syllectogram measuring unit. The sample was withdrawn into a glass cell (cross-sectional inner dimension, 0.8 × 0.8 mm) by a tube pump. Two sets of LEDs with a wavelength of 870 nm and the silicon photodiodes were arranged on both sides of the glass cell facing each other in the horizontal direction (Fig 1). The distance between the two detectors was about 9 mm, and the photometric hole diameter was 0.5 mm. By checking the difference between the two detectors, the effect of bubbles accidentally trapped in one of the photometric paths can be eliminated. The blood sample was warmed to 37°C and moved back and forth in the glass cell at a flow rate of 30 μL/s by the tube pump for erythrocyte dispersal. To measure erythrocyte aggregation, the red blood cells must be loaded with a sufficient shear rate to disperse them completely. Previous studies have shown that the mean shear rate required for the dispersion of red blood cells is about 200 s^−1^ and that the shear rate at which red blood cells noticeably deform is more than 1000 s^−1^ [37]. The shear rate used in the present experiment was approximately 335 s^−1^, which is sufficiently large compared with the conditions used in previous similar experimental systems [38]. The LED light transmitted through the blood sample was recorded through 12-bit analog-digital conversion at 10-ms intervals for 2 min by abruptly stopping the blood flow with a pinch valve. The aggregation parameters obtained using the two detectors were averaged. At the same time, using the complete blood count (CBC) measuring unit, which was similar to the performance-evaluated product [39], the total blood cell count including the Ht was measured using the remaining 20-μL samples, and the difference was confirmed to be within ±0.01 of the target value of the Ht. All measurements from sample aspiration were performed in triplicate.

### Determination of aggregation parameters

As shown in Fig 1, the time at which the transmitted light intensity becomes the minimum value (I_MIN_) after sudden flow cessation was defined as the starting time (t = 0). The AI was defined as a ratio of area A to the total of areas A and B. Area A is defined as the enclosed area between the syllectogram and minimum (I_MIN_). Area B is defined as the area between the syllectogram and transmitted light intensity at the end of the measurement (I_MAX_). AI was calculated as AI_5_, AI_10_, AI_30_, AI_60_, and AI_120_ from the syllectogram with measurement times of 5, 10, 30, 60, and 120 s, respectively.

## Results and discussion

### Correlation between the sedimentation and aggregation parameters

**Fig 2:**
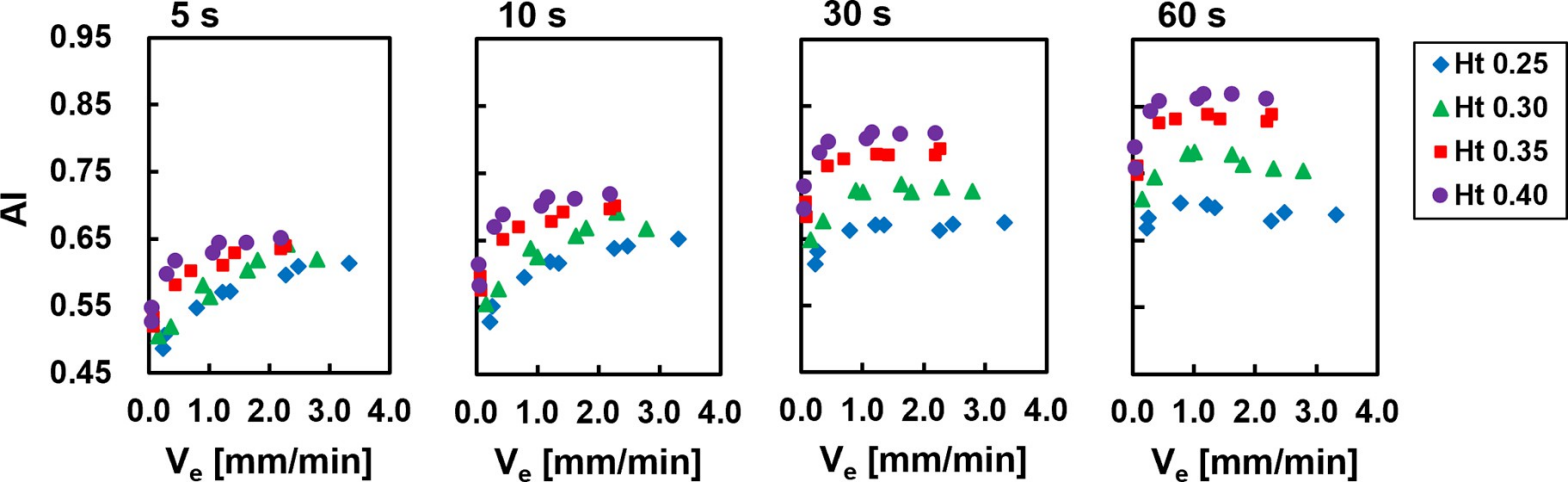
Relationship between the Sedimentation Velocity (V_e_) and AI for each measurement time at each Ht.

Fig 2 shows the relationship between the sedimentation velocity and the AI at four measurement times (5, 10, 30, and 60 s). The AI with a measurement time of 120 s was excluded because the reliability of the syllectogram fell with a significant increase in the transmitted light intensity from erythrocyte sedimentation (S1 Fig). At measurement times of 5 and 10 s, the AI showed a greater than 20% increase as the sedimentation velocity increased for each Ht. This result is similar to that of a previous report [18] and means that the higher the AI, a parameter reflecting the erythrocyte aggregation rate, the larger the erythrocyte aggregation size, leading to a higher sedimentation rate. The AI tended to increase by 20% or 40% as the measurement time increased but, when the measurement time was 30 and 60 s, the AI remained nearly constant at *V*_*e*_ > 1 mm/min. The correlation coefficients of the sedimentation parameters (WG ESR_1h_, *V*_*e*_, *α*, and *λ*) with the AI at each measurement time for each Ht are shown in Table 1. The correlation coefficients between the WG ESR_1h_ and the AI at each Ht tended to increase with shorter measurement times but were not particularly high even at a 5-s measurement time. This result shows that it is difficult to estimate the WG ESR_1h_ by using the AI alone because the Ht significantly affects the AI and *V*_*e*_. An AI less than the measurement time of ≤10 s for each Ht showed strong positive correlations for *V*_*e*_ and *α*. The correlation coefficients for the time constant at each Ht were slightly negative for an AI with all measurement times at Ht values of 0.35 and 0.40 but highly negative for an AI with 5-s and 10-s measurement times at Ht values of 0.25 and 0.30.

**Table 1:** Correlation coefficients between the sedimentation and AI.

		AI_5_	AI_10_	AI_30_	AI_60_
**WG ESR**_**1h**_	** **	0.697	0.551	0.119	−0.081
**Sedimentation velocity V**_**e**_	**Ht 0.25**	0.934	0.909	0.729	0.081
	**Ht 0.30**	0.924	0.902	0.739	0.349
	**Ht 0.35**	0.905	0.875	0.781	0.711
	**Ht 0.40**	0.844	0.812	0.748	0.709
**Size parameter α**	**Ht 0.25**	0.979	0.964	0.823	0.207
	**Ht 0.30**	0.962	0.954	0.844	0.504
	**Ht 0.35**	0.967	0.948	0.880	0.829
	**Ht 0.40**	0.933	0.909	0.860	0.832
**Time constant λ**	**Ht 0.25**	−0.972	−0.979	−0.899	−0.357
	**Ht 0.30**	−0.753	−0.717	−0.551	−0.236
	**Ht 0.35**	−0.351	−0.300	−0.160	−0.073
	**Ht 0.40**	−0.426	−0.377	−0.331	−0.319

Vertical rows show the WG ESR_1h_, the erythrocyte sedimentation velocity *V*_*e*_, size parameter *α*, and time constant *λ* at each Ht, and horizontal columns show the aggregation indices at measurement times of 5, 10, 30, and 60 s, respectively.

We believe the reason why the AI and the relationship between the AI and sedimentation parameters were affected by measurement time to be the following. The change in the transmitted light intensity within 10 s, which is greatly affected by *rouleaux* and aggregation formation, is significant [20, 21], but after 30 s, the aggregation speed slows down and the change in transmitted light intensity becomes tiny. As a result, the AI increased with a measurement time of 30 s or more while the sensitivity to the sedimentation velocity decreased because the amount of information on *rouleaux* and aggregation formation in the initial stage is relatively attenuated. Based on these results, we considered 5 s, which showed the strongest correlation with sedimentation parameters, to be the optimal measurement time for ESR estimation.

### Effect of the hematocrit on the aggregation index

**Fig 3:**
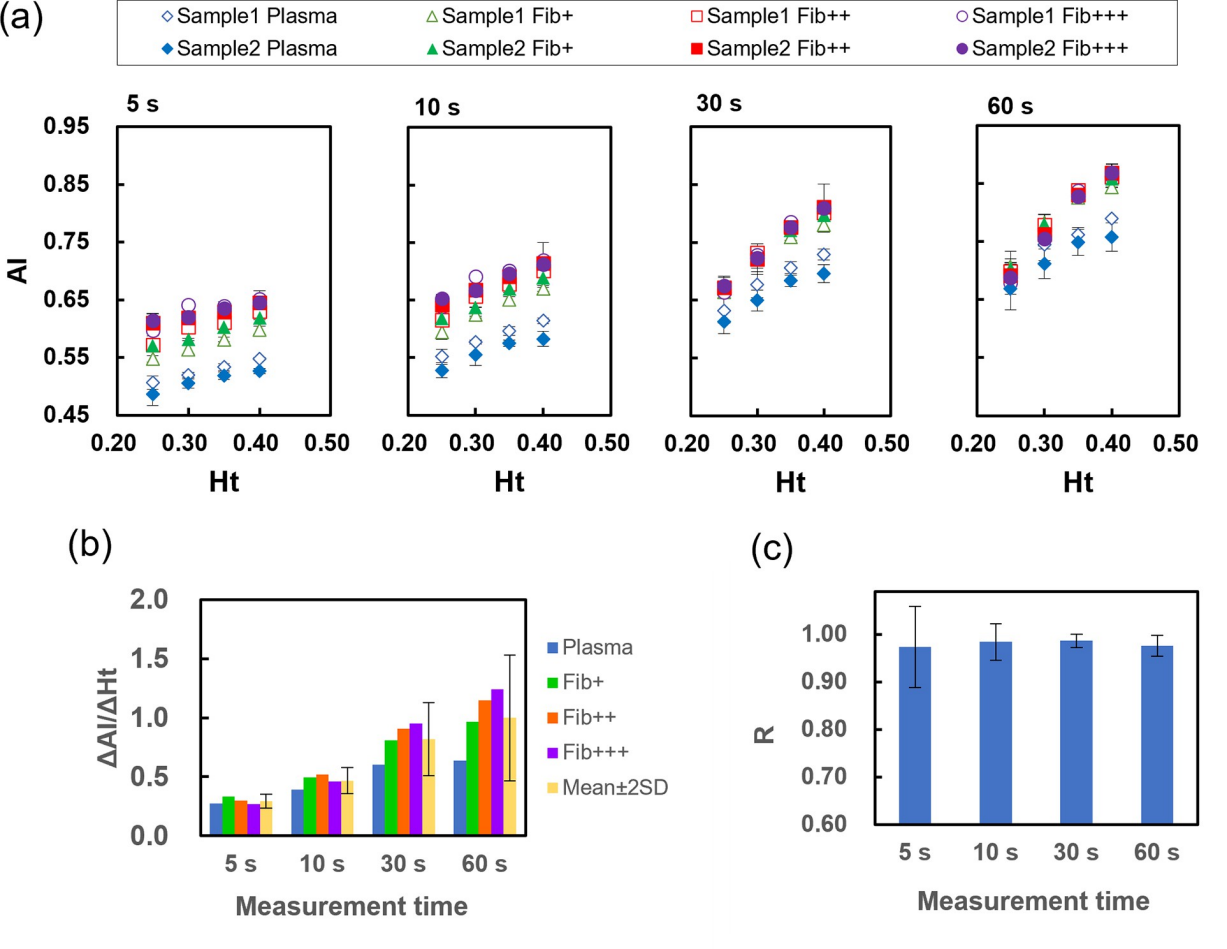
Analysis of the dependence of the Ht on the AI for each measurement time. (**a**) Relationship between the AI and Ht for each sample at each measurement time. Error bars indicate 2SD (n = 3). (**b**) The slope of the increase in the AI with the increase in the Ht (ΔAI/ΔHt) for each fibrinogen concentration at each measurement time. Error bars indicate 2SD among fibrinogen concentrations. (**c**) Average of the correlation coefficient between the AI and Ht for each sample. Error bars indicate SD (n = 4).

As shown in Fig 3A, the AI increased as the Ht increased at all measurement times. Such a Ht dependence of the AI was similar to that previously reported [20]. From Fig 2 and Table 1, it is clear that the AI reflects the size of the erythrocyte aggregate during settling at a fixed Ht. However, a high Ht increased the AI at the same fibrinogen level, which appears to contradict the view that a high Ht decreases the size of erythrocyte aggregates [10]. These facts suggest that the Ht shortens the convergence time of erythrocyte aggregate formation and increases the apparent AI. Fig 3B shows the slope of the increase in the AI for the increase in the Ht (ΔAI/ΔHt) at each measurement time. The ΔAI/ΔHt was less affected by the fibrinogen concentration and was close to constant at 5 and 10 s but was greatly affected at 30 and 60 s. The correlation coefficients between the AI and Ht at each measurement time were very high at all measurement times, as shown in Fig 3C, indicating that the AI increased almost linearly with the Ht. Considering this result and the fact that the correlation coefficient between sedimentation parameters and the AI shown earlier was highest at 5 s, the Ht-corrected aggregation index (HAI) was defined as the corrected AI for Ht 0.40, as expressed in Eq (15).


HAI=AI−k(Ht−0.40) (15)

Here, *k* is the correction factor depending on experimental conditions and *k* = 0.284, obtained as the average value of ΔAI/ΔHt at each fibrinogen concentration. The conventional AI includes the effect of plasma proteins as well as the Ht, so we cannot distinguish between them, but the HAI allows us to compare erythrocyte aggregability with various Ht values.

### Relationship between sedimentation parameters and aggregation parameters

**Fig 4:**
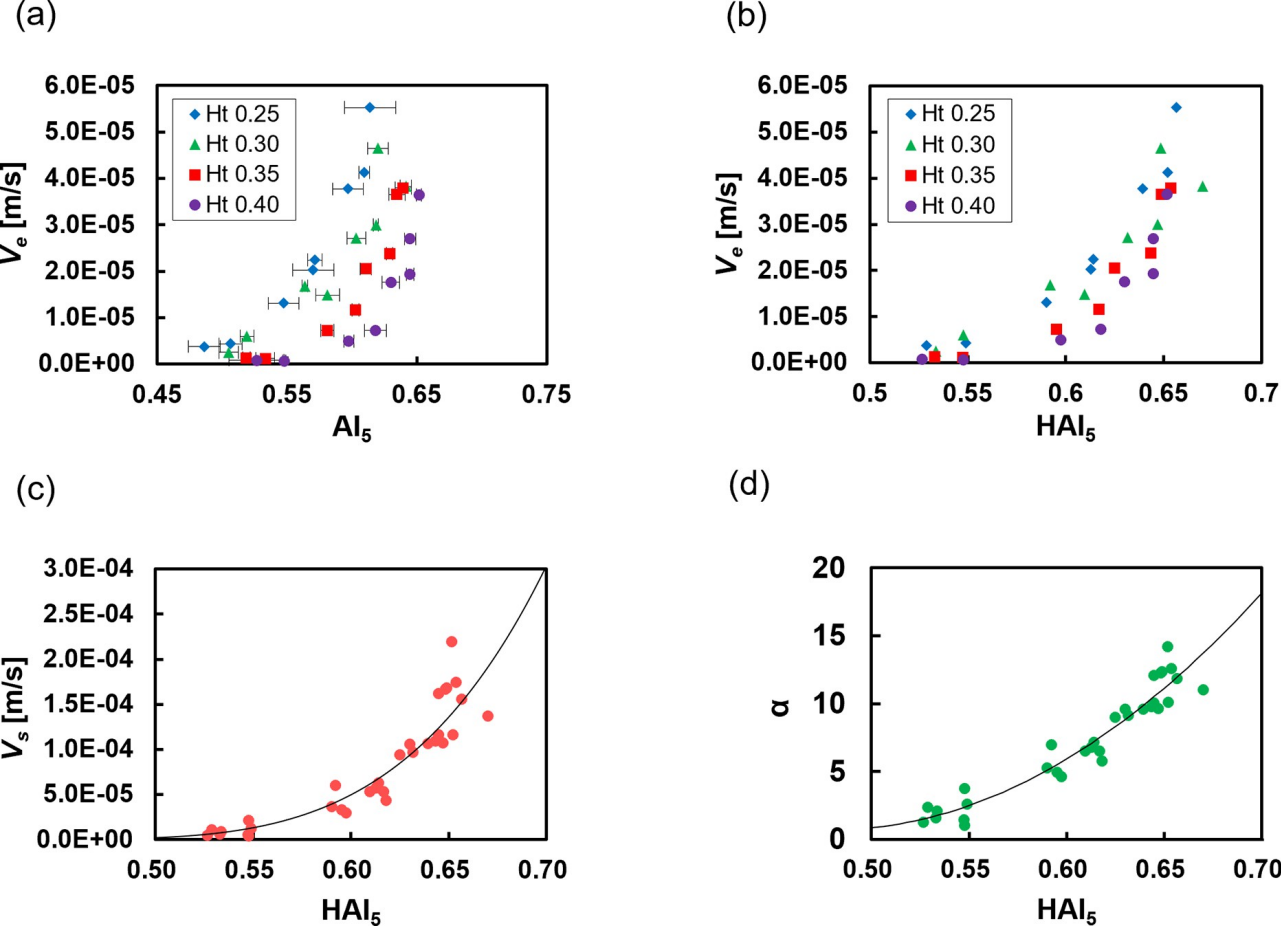
Analysis of the relationship between the sedimentation parameters and the aggregation parameters. (**a**) Relationship between the AI obtained from a 5-s measurement and the erythrocyte sedimentation; the solid line shows the guide. Error bars indicate 2SD (n = 3). (**b**) Relationship between the HAI obtained from a 5-s measurement and the erythrocyte sedimentation velocity. (**c**) Relationship between the HAI obtained from a 5-s measurement and the sedimentation velocity of an erythrocyte aggregate divided by the hindered settling effect. The solid line indicates the regression line. (**d**) Relationship between the HAI obtained from a 5-s measurement and the size parameter *α*. The solid line indicates the regression line.

The sedimentation velocity increased exponentially as the AI_5_ increased for each Ht, as shown in Fig 4A. Because the AI_5_ is affected by the Ht, the relationship of *V*_*e*_ to the HAI obtained with a 5-s measurement (HAI_5_) shifts in the positive direction of the x-axis with an increased Ht. However, the relationship of *V*_*e*_ to the HAI_5_ shown in Fig 4B indicates that the apparent increase in the AI due to the Ht is corrected and that the horizontal shift is canceled. Interestingly, as shown in Fig 4C, the sedimentation velocity eliminating the hindered settling, that is, the sedimentation velocity of a single aggregate (*V*_*s*_), increased in a power law function with the HAI_5_ as a variable, independently of the Ht. Furthermore, as shown in Fig 4D, the size parameter *α* was in good agreement with the quadratic function of the HAI_5_ (*r*^2^ = 0.913). These results suggest that the sedimentation velocity of a single aggregate could be expressed by the HAI_5_, a parameter that reflects the aggregate size. Here, we described *V*_*s*_ by the regression equation of the quartic function of the HAI_5_ as Eq (16) because *V*_*s*_ is proportional to the square of *α*.


$$V_{s}={a({HAI}_{5}-b)}^{4}+c$$ (16)

**Fig 5:**
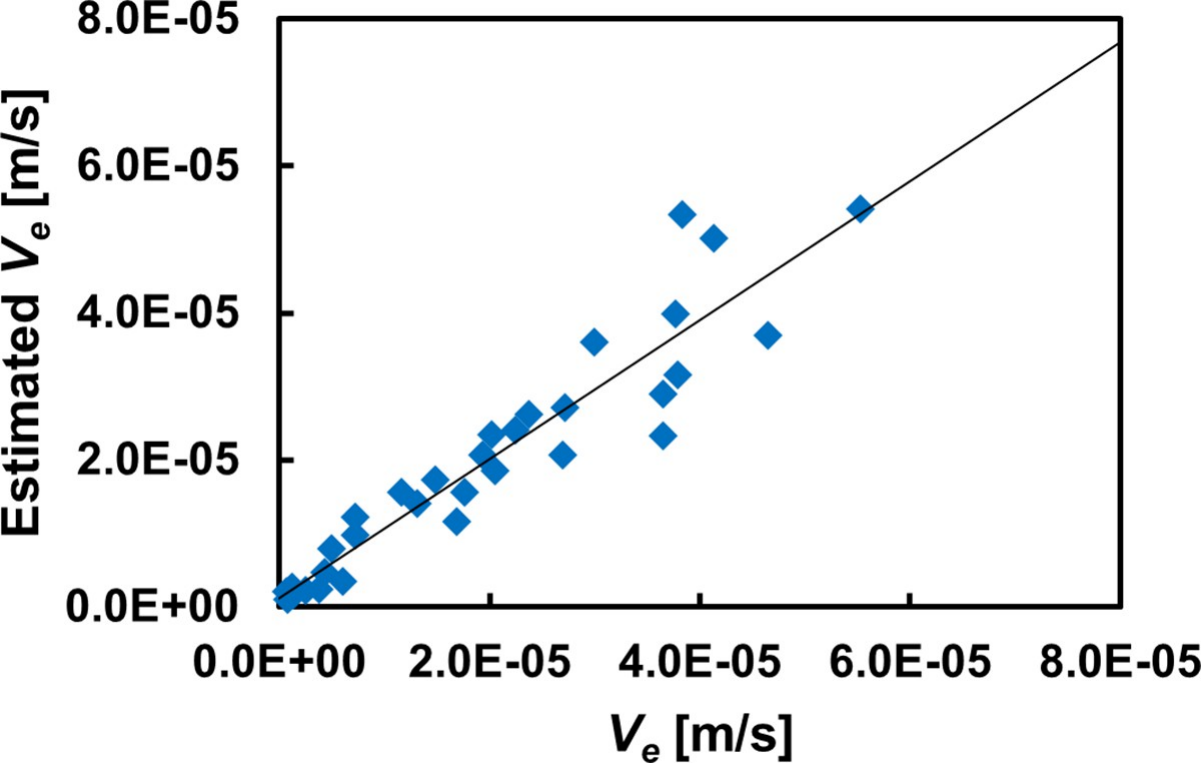
Comparison between the sedimentation velocity obtained from the Westergren method and the value estimated by the regression equation. Correlation coefficient, *r* = 0.940 (n = 32). The solid line indicates the regression line.

Here, *a* is the coefficient, *b* is the minimum physiological value of the HAI_5_, and *c* is the sedimentation velocity in the absence of aggregation, that is, the sedimentation velocity determined by Eq (2). *a* and *b* were determined by data fitting (*a* = 0.0541, *b* = 0.426). *V*_*s*_ was obtained using this regression equation, and the sedimentation curve was calculated by determining the calculated values of *V*_*e*_ and *α*. Fig 5 shows the comparison between the *V*_*e*_ estimated using our method and the *V*_*e*_ obtained from the measured sedimentation curve. The estimated *V*_*e*_ showed a very high correlation (*r* = 0.940, *p* < 0.001) with the measured values. These results indicate that the Ht-corrected AI can be integrated into the sedimentation theory based on the modified Stokes’ law and is useful for estimating *V*_*e*_ with high accuracy. Incidentally, the WG ESR_1h_ divided by the measurement time and adjusted to the unit of *V*_*e*_ had an almost linear relationship with the *V*_*e*_ calculated from the sedimentation curve (*r* = 0.990), with a slope of 0.778. Hence, this relationship can be used in future evaluations to simply determine *V*_*e*_ from WG ESR_1h_.

**Fig 6:**
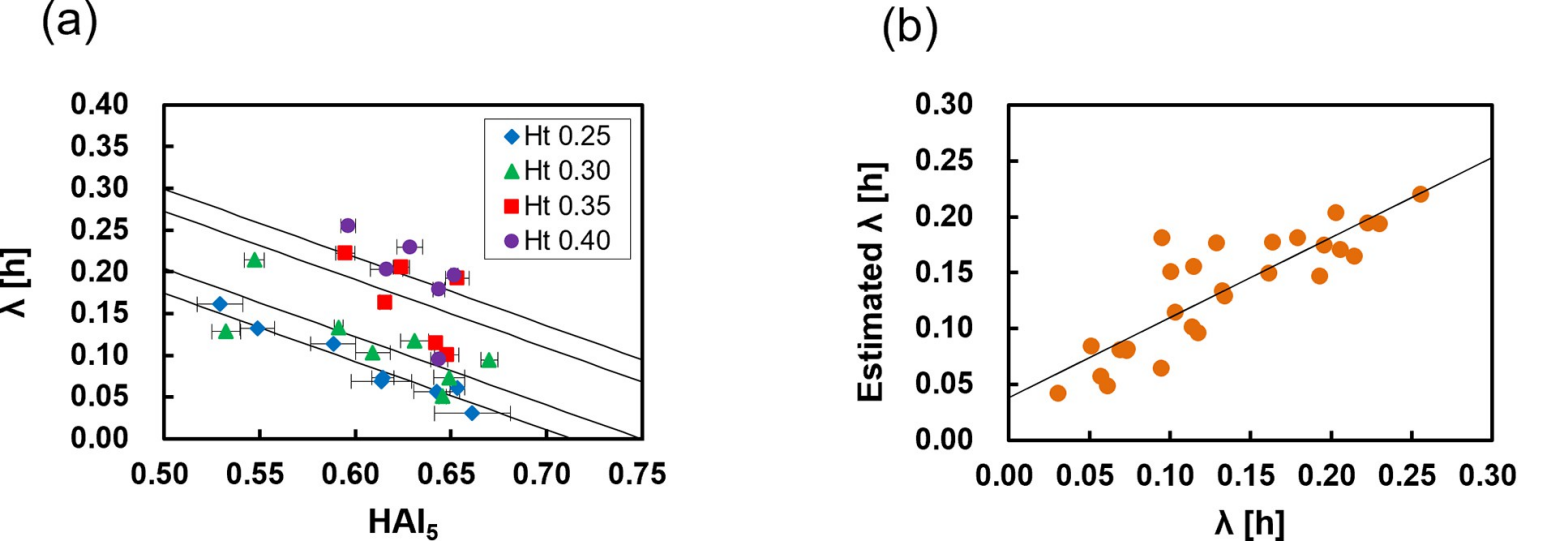
Analysis and estimation of the time constant of the sedimentation curve. (**a**) Relationship between the HAI obtained from a 5-s measurement and the time constant *λ*; the solid line shows the guide. Error bars indicate 2SD (n = 3). (**b**) Comparison between the time constant obtained from the sedimentation curve and the value estimated by the regression equation. The solid line indicates the regression line. Correlation coefficient, *r* = 0.851 (n = 28).

The relationship between the HAI_5_ and *λ* is shown in Fig 6. Unlike the results for *V*_*e*_, *λ* decreased almost linearly with the HAI_5_ at Ht values of 0.25 and 0.30. However, for Ht values of 0.35 and 0.40, the estimated *λ* varied greatly due to the low reliability of the data fitting caused by the very slow sedimentation rate. The reason why *λ* decreased with an increase in the HAI_5_ is that the higher the erythrocyte aggregation rate, the faster the sedimentation start time, as shown in S2 Fig. Moreover, for a very high HAI, the settling speed did not decrease until near the end. This may be because large erythrocyte aggregates settled quickly and were densely packed in the bottom of the tube, and the Ht near the sedimentation surface did not significantly increase [6, 29], resulting in the decrease in settling speed occurring later due to the increase in the hindered settling effect being delayed until the end. We assumed that the relationship between the AI and *λ* was linear and that the slope was constant regardless of the Ht, based on Fig 6A, and attempted to estimate *λ* by the regression equation *λ* = *dHAI*_5_+*eHt*+*f*, where *d*, *e*, and *f* are the coefficients. *d* was obtained as the average value of the slopes of *λ* for the HAI_5_ at Ht values of 0.25 and 0.30 and the coefficients *e* and *f* were determined by data fitting (*d* = −0.816, *e* = 0.887, *f* = 0.357). Fig 6B shows a comparison between the *λ* obtained by the regression equation and the *λ* obtained from the measured sedimentation curve. The estimated *λ* shows a high correlation with the measured value (*r* = 0.851, *p* < 0.001).

### Comparison of the estimated ESR and measurement value

**Fig 7:**
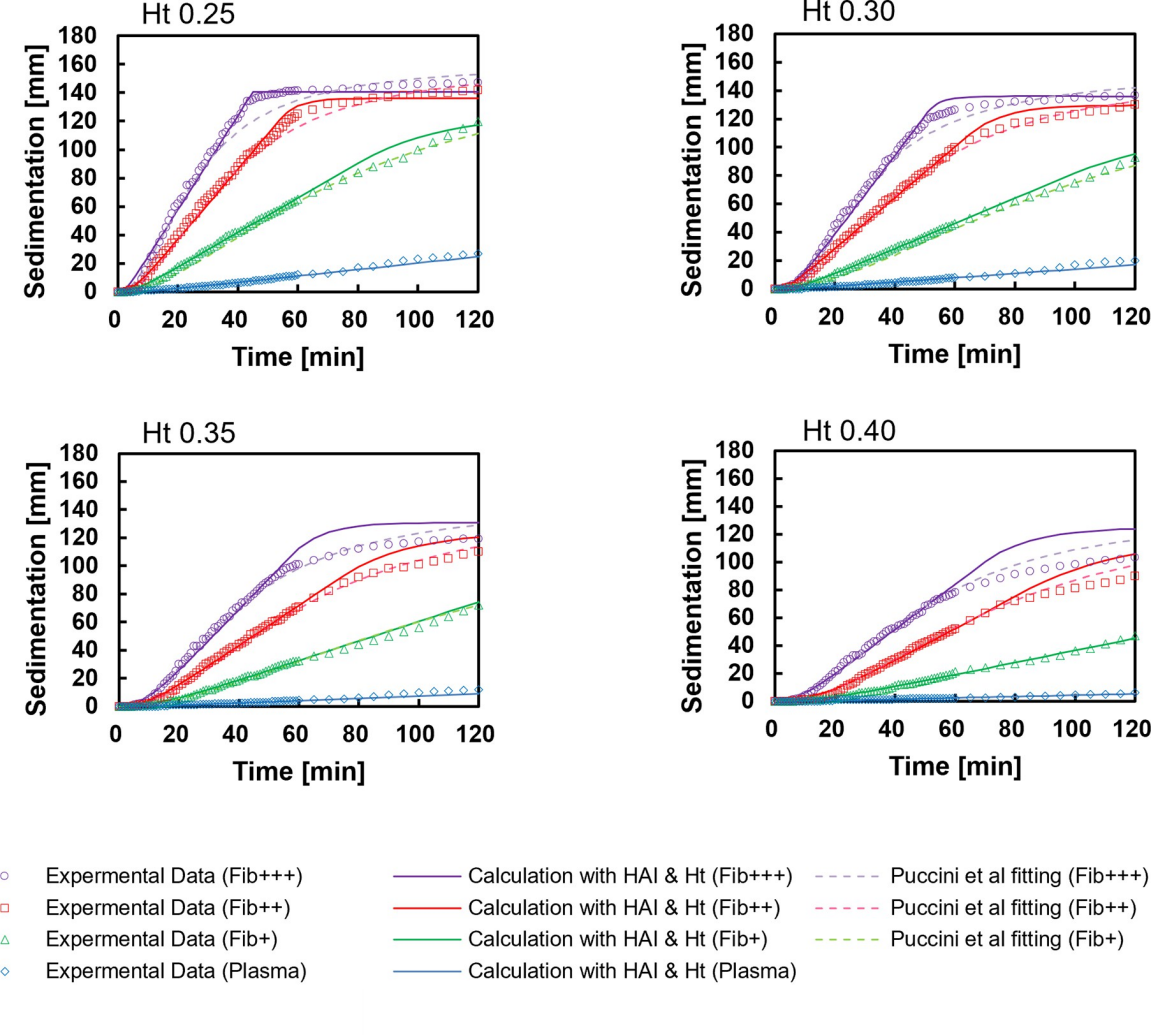
Comparison of representative sedimentation curves for each sample. The plots indicate experimental data. The solid lines indicate the sedimentation curves calculated with the HAI and Ht. The dotted lines indicate Puccini’s fitting curve.

**Fig 8:**
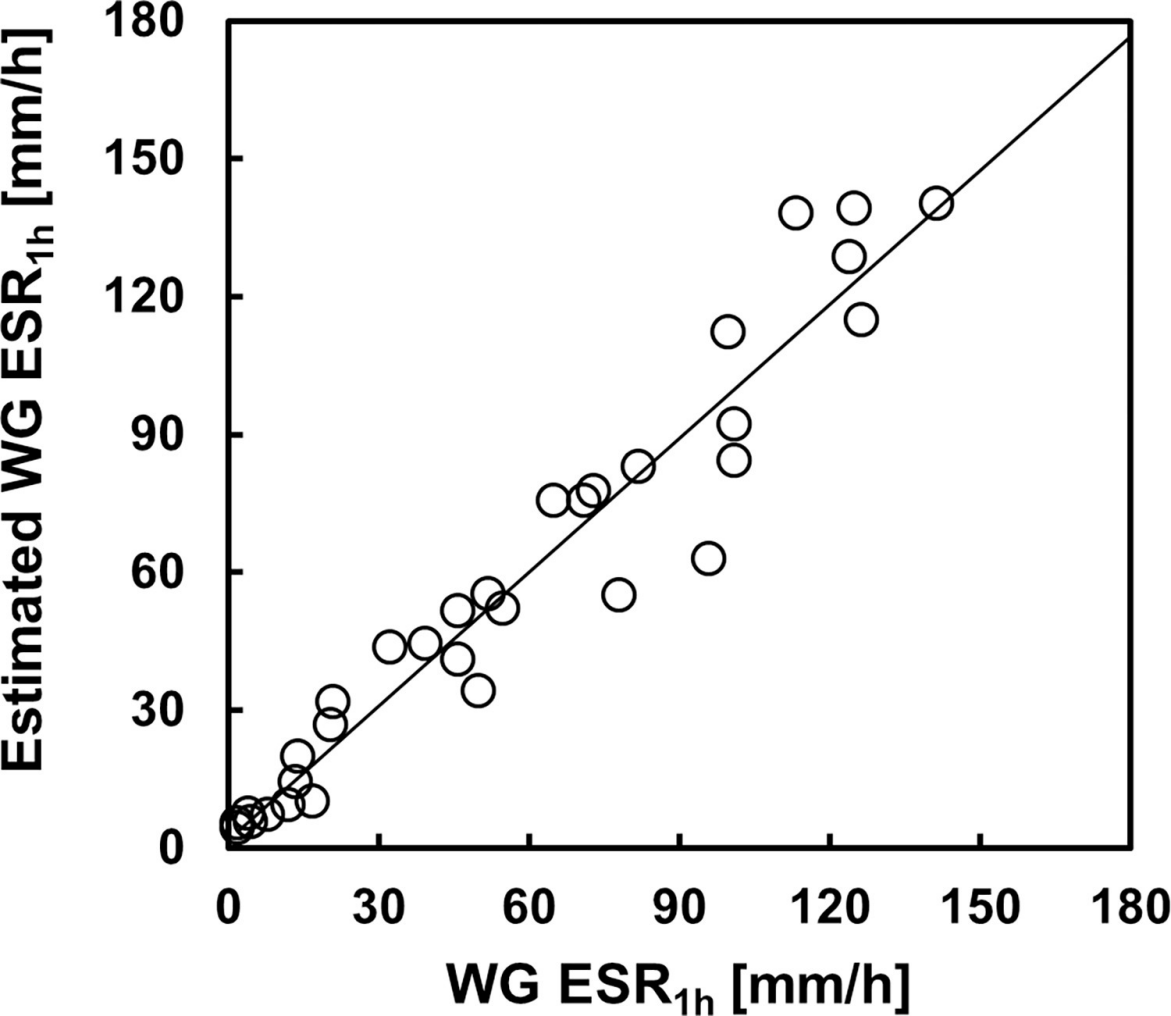
Relationship between the ESR at 60 min obtained by the Westergren method and the estimated value. Correlation coefficient, *r* = 0.966 (n = 32). The solid line indicates the regression line.

Fig 7 shows a comparison of the sedimentation curve observed by the Westergren method, the sedimentation curve calculated with the HAI_5_ and Ht, and the fitting curve of Puccini *et al*. [30]. At a Ht of 0.25, the sedimentation curve obtained with our method almost matched the measured value from the start to 60 min. However, Puccini’s fitting curve was lower than the measured value near the transition time from the constant sedimentation rate phase to the packing phase at a Ht of 0.25. In contrast, for Ht values of 0.35 and 0.40, the values estimated by our method were higher than those measured after 60 min. We assume that the coefficient of the equation for the transition time was not optimal for our conditions. Indeed, we confirmed that the agreement could be improved by adjusting the coefficients. Alternatively, as in previous work, further improvement may be possible if the decrease in the sedimentation velocity caused by the increase in the Ht on the sedimentation surface during the packing phase is accurately expressed [29]. However, our method can estimate with sufficiently high accuracy the sedimentation distance after 60 min, which is necessary for clinical examination. As shown in Fig 8, the correlation between the ESR at 1 h obtained by our method and the measured values was excellent (*r* = 0.966, *p* < 0.001).

We successfully demonstrated that our method could accurately estimate the sedimentation curve for at least 60 min by measuring erythrocyte aggregation for only 5 s, which is shorter than some proposed methods [18, 29]. Moreover, our method can be applied to blood samples with a wide range of fibrinogen concentrations and Ht values. In addition, the device can simultaneously measure the syllectogram and CBC in a few minutes, making it suitable for our ESR estimation methods that require the Ht. The clinical performance of a rapid ESR measuring system using a similar method had been evaluated prior to this report [40]. In the present study, we clarified the details of the method for calculating the sedimentation rate using the HAI with the optimal measurement time and modified Stokes’ law. Although this experiment focused solely on the effect of Ht on erythrocyte aggregation by varying only Ht and fibrinogen, the limited number of subjects should be considered a limitation of this experiment because ESR is strongly affected by age, sex, and disease [36]. Our next step will be to show the validity of this method in a large number of blood samples from individuals with a wide range of clinical backgrounds. Because there are many opportunities to test both the ESR and CBC as hematology tests, our simultaneous ESR and CBC measurement system offers a considerable advantage in the clinical setting. When a medical practitioner measures the ESR and CBC, it is usually necessary to collect a few milliliters of blood in dedicated tubes and measure them separately. However, with our method, only 80 μL of blood from one EDTA tube needs to be measured once, which will reduce the burden on patients and improve the efficiency and throughput of the clinical workflow.

## Conclusion

In this study, we investigated the effects of measurement time and Ht on the erythrocyte AI in order to ESR in a short time. The correlation between the AI and the sedimentation velocity and time constant in the sedimentation curve for each Ht was very high at measurement times of 5 and 10 s. Furthermore, the AI increased almost linearly with an increase in the Ht and was not significantly affected by the fibrinogen concentration at these measurement times. We defined the HAI (Ht-corrected aggregation index) using the AI at a 5-s measurement time and the Ht. The sedimentation velocity of a single aggregate calculated by eliminating the hindered settling effect was in good agreement with the exponential function of the HAI, and the time constant of the sedimentation curve could also be described by the linear regression equation of the HAI and Ht. The ESR value calculated based on the modified Stokes’ law and HAI showed an excellent correlation with the results from the Westergren method.

## Supporting information

S1 Fig Syllectogram from a representative measurement of a fibrinogen-spiked sample (Ht 0.25, Ht 0.30, Ht 0.35, Ht 0.40). (TIF)

S2 Fig Rescaled representative sedimentation curves for each sample. These graphs show a modified scale of Fig 7, expanded from 0 min to 20 min. The plots indicate experimental data. The solid lines indicate the sedimentation curves calculated with the HAI and Ht. The dotted lines indicate Puccini’s fitting curve.
(TIF)

S1 Dataset These data sets represent minimal data set for this study. (XLSX)
