# Supplementary figures and images for: A rapid and accurate method for estimating the erythrocyte sedimentation rate using a hematocrit-corrected optical aggregation index
Source: PLoS One. 2022 Jul 12;17(7):e0270977. doi: 10.1371/journal.pone.0270977 (PMC9275726; doi:10.1371/journal.pone.0270977)

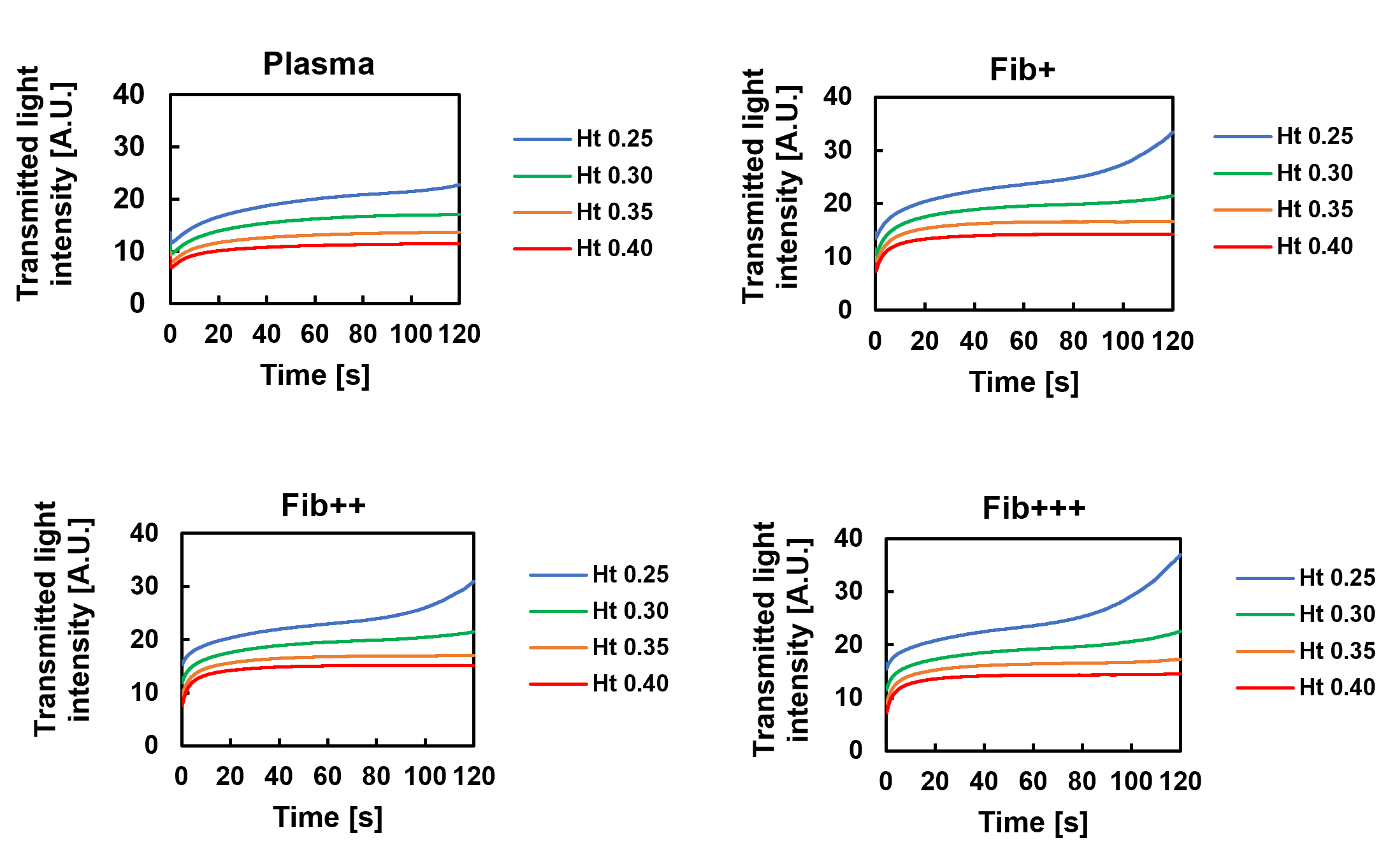

Supplement: S1 Fig — (TIF) [file pone.0270977.s001.tif]

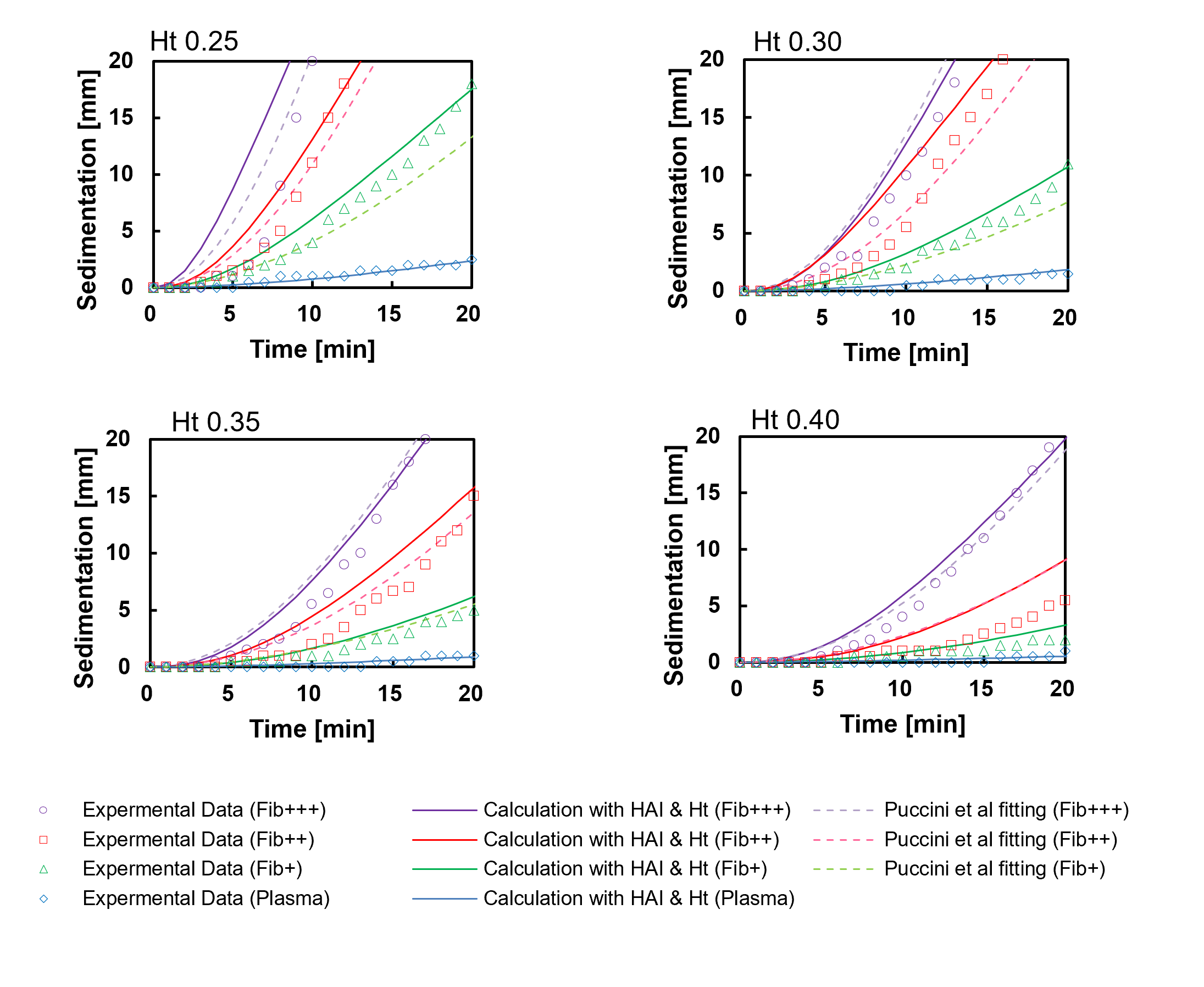

Supplement: S2 Fig — These graphs show a modified scale of Fig 7, expanded from 0 min to 20 min. The plots indicate experimental data. The solid lines indicate the sedimentation curves calculated with the HAI and Ht. The dotted lines indicate Puccini’s fitting curve. (TIF) [file pone.0270977.s002.tif]
